# Bibliometric and visual analysis of cardiovascular diseases and COVID-19 research

**DOI:** 10.3389/fpubh.2022.1022810

**Published:** 2022-12-08

**Authors:** Namin Wei, Yan Xu, Huan Wang, Qiulei Jia, Xintian Shou, Xuesong Zhang, Nan Zhang, Ya'nan Li, Huaqiang Zhai, Yuanhui Hu

**Affiliations:** ^1^Standardization Research Center of Traditional Chinese Medicine Dispensing, School of Chinese Materia Medica, Beijing University of Chinese Medicine, Beijing, China; ^2^Department of Cardiovascular Diseases, Guang'anmen Hospital, China Academy of Chinese Medical Sciences, Beijing, China

**Keywords:** cardiovascular diseases, COVID-19, CiteSpace, bibliometrics, visualization

## Abstract

**Background:**

The global community has been affected by the coronavirus disease 2019 (COVID-19), which emerged in December 2019. Since then, many studies have been conducted on cardiovascular diseases (CVDs) and COVID-19. The aim of this study was to perform a bibliometric and visual analysis of the published relationship between CVDs and COVID-19.

**Methods:**

1,890 publications were retrieved from the Web of Science Core Collection database on January 5, 2022. Microsoft Office Excel and CiteSpace were then used to carry out scientometric analysis on the relevant literature according to seven aspects: document type, countries/regions, institutions, authors, journals, references, and keywords.

**Results:**

The research on CVDs and COVID-19 is currently in a period of rapid development, with China, USA, England, and Italy leading the field. There is active cooperation between most countries and institutions. Harvard Medical School stands out among the many institutions not only for the largest number of publications, but also for their high quality. Banerjee A, Solomon SD and Narula J are three representative authors in this field. *Frontiers in Cardiovascular Medicine* was the journal with the highest number of published studies, and *The Lancet* was the most cited journal. Two documents with a high degree of significance in this field were identified. Popular research topics in this field are specific diseases, such as acute coronary syndrome and heart failure; pathogenesis related to ACE2, insulin resistance and pericyte; the specific therapeutic drug chloroquine; and clinical characteristics, physical activity, and mental health. ACE2 and NF-κB will be the focus of future research.

**Conclusions:**

This study provides useful information for the research of CVDs and COVID-19, including potential collaborators, popular research topics, and a reference for more extensive and in-depth research in the future.

## Introduction

The coronavirus disease 2019 (COVID-19) is caused by the novel Severe Acute Respiratory Syndrome Coronavirus type 2 (SARS-CoV-2). Ever since the first case of COVID-19 was detected in Wuhan, China in December 2019, both the global health system and the whole world have faced severe challenges (1). In addition to Severe Acute Respiratory Syndrome Coronavirus (SARS-CoV) (2) in 2003 and Middle East Respiratory Syndrome Coronavirus (MERS-CoV) (3) in 2012, SARS-CoV-2 is the third high-risk virus to be found in humans. It is an enveloped positive single-stranded RNA virus (4), characterized by low pathogenicity and high transmissibility (5). On March 11, 2020, the World Health Organization (WHO) declared COVID-19 as a global pandemic. As of February 6, 2022, more than 392 million confirmed cases and over 5.7 million deaths have been reported globally (6).

As the COVID-19 pandemic enters its third year, although it is known that the lungs are the main organ involved in COVID-19 infections, SARS-CoV-2 also causes systemic diseases with multiple clinical manifestations (7). The core pathology of COVID-19 is the infection of respiratory cells by SARS-CoV-2, resulting in excessive inflammation and respiratory diseases. The most serious cases involve cytokine storm and acute respiratory distress syndrome (ARDS) (1). Angiotensin-converting enzyme 2 (ACE2) is the host cell receptor for the viral spike protein through which SARS-CoV-2 infects the heart, vascular tissues and circulating cells (8). Specific cardiovascular diseases (CVDs) may occur including acute myocardial injury and myocarditis (9, 10), arrhythmia (11, 12), cardiac fibrosis (13), heart failure (HF) (14, 15), vascular dysfunction (16), and thromboembolic diseases (17). Among the confirmed COVID-19 cases reported by the National Health Commission of China (NHC), some patients were initially admitted to the hospitals due to cardiovascular symptoms (11). Several studies have reported that the presence of CVDs and its risk factors are associated with severe COVID-19 and higher mortality (18–20). In addition, many antiviral drugs can also cause cardiac insufficiency, arrhythmias, or other CVD during COVID-19 treatment (21, 22). The COVID-19 pandemic has led to severe disruptions in health care service around the world, and the global reduction in cardiovascular diagnostic testing had a significant impact on overall CVDs morbidity and mortality (23, 24). Widespread social lockdowns and restructuring of healthcare systems can also lead to less-than-optimal treatment of patients with acute or established CVDs (25).

Bibliometrics is a discipline that studies the distribution structure, quantitative relationships, rules of change and quantitative management of document information using mathematical, statistical and other metrological research methods (26). Bibliometrics differs from traditional literature reviews by allowing for quantitative and qualitative analysis of the literature to assess the current status, trends, and frontiers of research activities (27, 28). In addition, this method compares the contributions of different countries/regions, institutions, journals, and scholars (29, 30). A unique feature of a high-level bibliometric study is that it will provide researchers with frontier areas of research to save time (31). Researchers commonly employ CiteSpace as a bibliometric visualization tool for data analysis and visualization (32, 33). Since the pandemic, researchers around the world have published many studies on CVDs and COVID-19. Previously, a bibliometric analysis on cardiac involvement in COVID-19 had used VOSviewer software for bibliometric analysis (34). In this study, the relevant CVDs and COVID-19 literature contained on the Web of Science Core Collection was reviewed and CiteSpace visualization software was used to perform bibliometric analysis on the relevant literature. Visual knowledge maps were drawn the research statuses were displayed and various popular research topics were identified, which will fill the gaps from previous papers and provide references for future research.

## Methods

### Data collection

**Figure 1:**
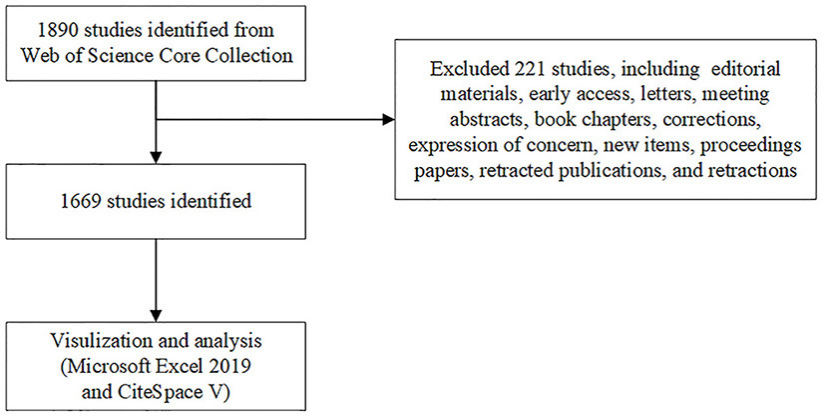
Flowchart of literature selection.

The following search terms were used to retrieve literature from the Web of Science Core Collection (http://apps.webofknowledge.com/) on January 5, 2022: Topic = (“COVID-19” OR “COVID 19” OR “SARS-CoV-2 Infection” OR “Infection, SARS-CoV-2” OR “SARS CoV 2 Infection” OR “SARS-CoV-2 Infections” OR “2019 Novel Coronavirus Disease” OR “2019 Novel Coronavirus Infection” OR “2019-nCoV Disease” OR “2019 nCoV Disease” OR “2019-nCoV Diseases” OR “Disease, 2019-nCoV” OR “COVID-19 Virus Infection” OR “COVID 19 Virus Infection” OR “COVID-19 Virus Infections” OR “Infection, COVID-19 Virus” OR “Virus Infection, COVID-19” OR “Coronavirus Disease 2019” OR “Disease 2019, Coronavirus” OR “Coronavirus Disease-19” OR “Coronavirus Disease 19” OR “Severe Acute Respiratory Syndrome Coronavirus 2 Infection” OR “SARS Coronavirus 2 Infection” OR “COVID-19 Virus Disease” OR “COVID 19 Virus Disease” OR “COVID-19 Virus Diseases” OR “Disease, COVID-19 Virus” OR “Virus Disease, COVID-19” OR “2019-nCoV Infection” OR “2019 nCoV Infection” OR “2019-nCoV Infections” OR “Infection, 2019-nCoV” OR “COVID19” OR “COVID-19 Pandemic” OR “COVID 19 Pandemic” OR “Pandemic, COVID-19” OR “COVID-19 Pandemics”) AND (“Cardiovascular Diseases” OR “Cardiovascular Disease” OR “Disease, Cardiovascular” OR “Diseases, Cardiovascular”) AND Language = English. The retrieval time was set from 2020 to 2022. The retrieved data were collected within 1 day to avoid any potential deviations due to daily updates. A total of 1,890 publications were retrieved, of which 221 irrelevant publications were excluded, including editorial materials, early access, letters, meeting abstracts, book chapters, corrections, expression of concern, new items, proceedings papers, retracted publications, and retractions. A total of 1,669 publications were exported in the form of full records with cited references, saved as plain text files, and stored in download_.txt format. Figure 1 shows the flowchart for literature selection.

### Data analysis

Microsoft Office Excel 2019 and CiteSpace (5.8.R3) were utilized to analyze all 1,669 publications and Microsoft Office Excel 2019 was used to construct the tables. CiteSpace (a web-based Java application) is a document visualization analysis software that was gradually developed for bibliometrics and data visualization (31). In this study, CiteSpace was employed to construct visual knowledge maps for the distribution of countries/regions, institutions, authors, journals, references, and keywords (35). The parameters of CiteSpace were set as follows: time slice (2020–2022), years per slice (1), term source (entire selection), node type (chosen one at a time), and selection criteria (top 100% objects). Other parameters were left at the default settings. Modularity is an index that is used to evaluate network modularity. The greater the modularity value of a network, the better the clustering of the network. The value space for Q was (0, 1), with Q > 0.3 indicating that the obtained network community structure is significant (36). The silhouette was evaluated by measuring the network homogeneity. The closer the silhouette value is to 1, the higher the network homogeneity. A clustering result with a value of 0.7 is highly reliable, while a result above 0.5 can be considered reasonable (36).

## Results

### Number of publications and distribution type

The number of publications within a period reflects the research development speed and trends in a discipline (37). A total of 1,890 publications related to CVDs and COVID-19 were identified in the Web of Science Core Collection database, reflecting the rapid development in CVDs and COVID-19 research during this period. Table 1 shows the types of publications that were analyzed, where 61.47% (1,026) of the documents were articles and 38.53% (643) were review articles. Among the publications, there were a total of 33,788 articles that were cited 54,277 times, with an h-index of 86, and an average per item of 32.52.

**Table 1:** Publication type.

**No**.	**Publication type**	**Total publications**	**Citing articles**	**Times cited**	**Average per item**	**% of 1,669**	**H-index**
1	Articles	1,026	27,043	37,449	36.50	61.47	64
2	Review articles	643	12,315	16,828	26.17	38.53	54

### Distribution of countries/regions and institutions

A total of 1,669 publications were published from 111 different countries/regions and 3,715 institutions. As shown in Table 2, the greatest number of publications came from USA (496, 29.72%), followed by China (249, 14.92%), Italy (234, 14.02%) and England (175, 10.49%). These four countries accounted for more than half of the total reports, which shows that USA, China, Italy, and England are the leading countries in CVDs and COVID-19 research.

**Table 2:** Top 10 countries/regions and institutions in CVDs and COVID-19 research.

**Rank**	**Countries/Regions**	**Record**	**BC**	**Count (%)**	**Institutions**	**Record**	**BC**	**Count (%)**
1	USA	496	0.00	29.72	Harvard Med Sch	81	0.15	4.85
2	China	249	0.00	14.92	UCL	58	0.02	3.48
3	Italy	234	0.00	14.02	Univ Calif System	49	0.07	2.94
4	England	175	0.04	10.49	Huazhong Univ Sci & Technol	43	0	2.58
5	Germany	112	0.04	6.71	Institut National de la Santé et de la Recherche Médicale	42	0	2.52
6	India	94	0.03	5.63	Wuhan Univ	39	0.05	2.34
7	Spain	89	0.00	5.33	Assistance Publique Hopitaux Pairs Aphp	32	0	1.92
8	Australia	80	0.05	4.79	Ciber Centro de Investigacion Biomedica en Red	31	0	1.86
9	Brazil	75	0.00	4.49	Sapienza Univ Rome	30	0	1.80
10	Canada	75	0.02	4.49	Univ Tehran Med Sci	30	0	1.80

BC, Betweenness Centrality.

**Figure 2:**
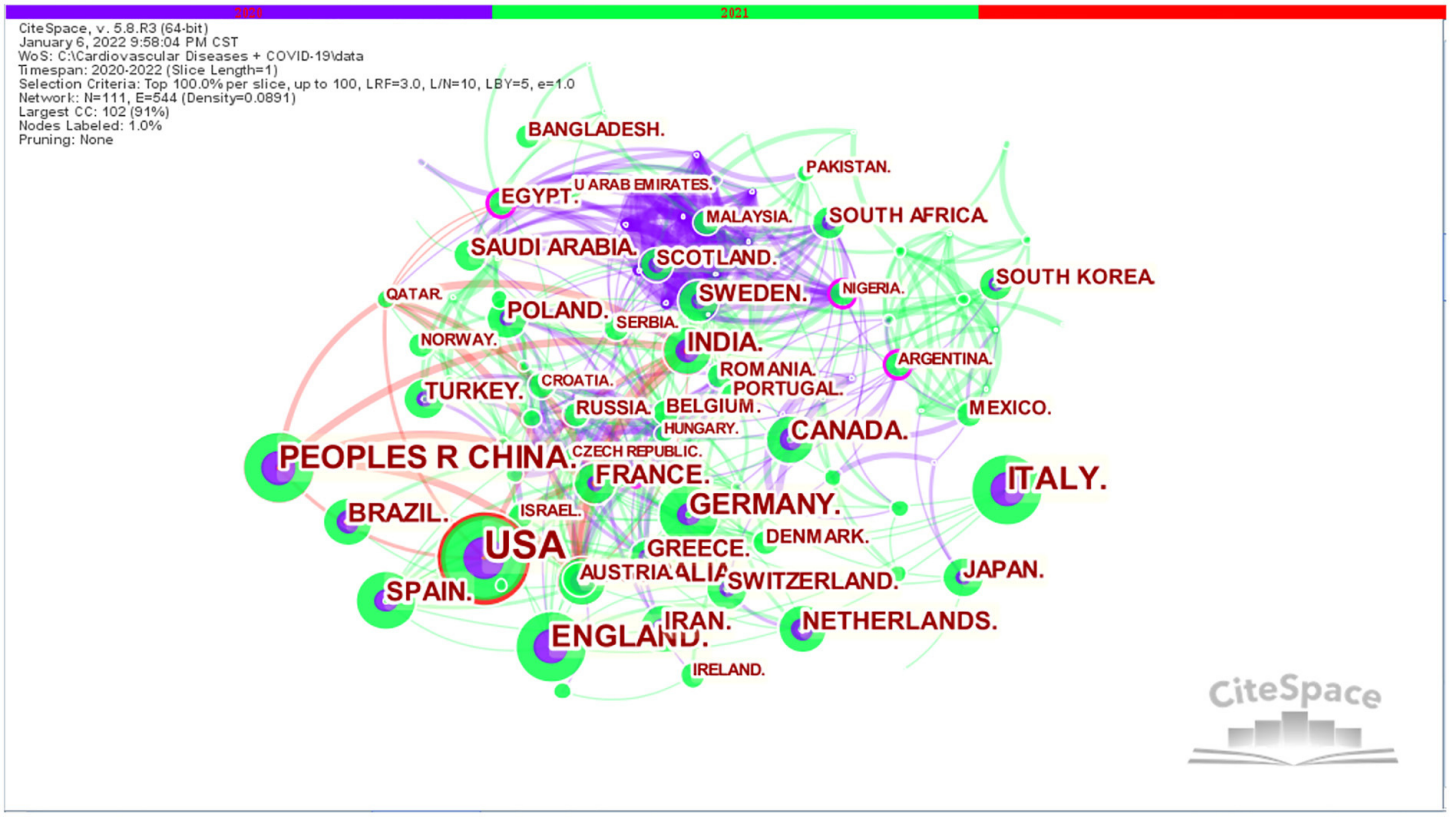
Visualization map of countries/regions in the studies of CVDs and COVID-19 research.

**Figure 3:**
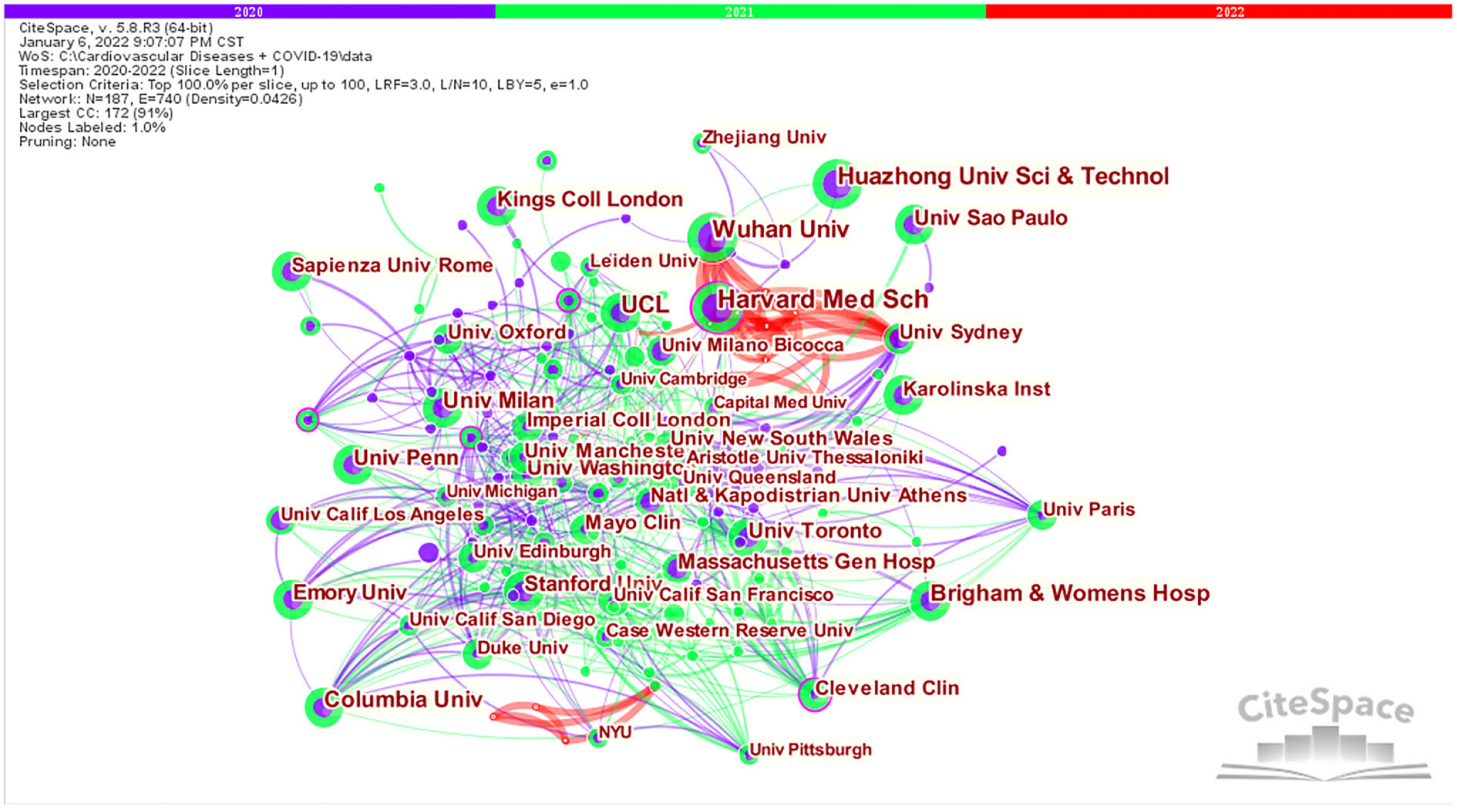
Visualization map of institutions in the studies of CVDs and COVID-19 research.

In CiteSpace, the cooperative scientific research networks of the different countries/regions, institutions and authors correspond to the macro, meso and micro cooperative networks, respectively. The visual knowledge maps can provide information to aid in the identification of influential research teams and potential collaborators, thus helping researchers establish cooperative relationships (38). As illustrated in Figures 2, 3, each circle represents a country/institution, and the size of the circle indicates the number of publications output by the country/institution. The lines between the circles denote cooperation between countries/institutions, with wider lines, closer cooperation. There was active cooperation between many countries and institutions, such as USA, China, India, Australia, Brazil, Harvard Medical School, Wuhan University, Huazhong University of Science and Technology, University College London, Zhejiang University, University of Sydney, and Milan Bicocca University.

In CiteSpace, the nodes with betweenness centrality > 0.1 are the key points (39). CiteSpace uses betweenness centrality to find and measure the importance of documents. A purple circle highlights such documents (or authors, journals, countries, and institutions) and indicates that the node betweenness centrality is > 0.1. Publications with high betweenness centrality are usually key hubs connecting two different fields, also known as turning points in CiteSpace (32). An analysis of the country distribution of these publications shows that Australia, England, and Germany have the highest betweenness centrality (Table 2), but none of them exceeded 0.1, indicating that influential countries have not yet been formed in this field. An analysis of the distribution of research institutions shows that Harvard Medical School is the most prominent research institution for co-authored papers, not only for the largest number of articles written in collaboration but also for the highest betweenness centrality (0.15), revealing that Harvard Medical School is highly outstanding in CVDs and COVID-19 research, both quantitatively and qualitatively.

### Distribution of authors

A total of 12,823 authors were involved in the publication of literature on CVDs and COVID-19 research. From Table 3, we can see that Banerjee A and Narula J's betweenness centrality is 0.02, Solomon SD's betweenness centrality is 0.01, and other authors' betweenness centrality is 0, which indicates that there are no influential authors in CVDs and COVID-19 research. The reason for this situation is probably due to the short 2-year time span.

**Table 3:** Top 10 authors in CVDs and COVID-19 research.

**Rank**	**Frequency**	**Author**	**Citations**	**BC**	**Institutions**	**H-index**
1	8	Banerjee A	480	0.02	University of London	28
2	7	Sliwa K	30	0.00	University of Cape Town	72
3	6	Solomon SD	1,026	0.01	Harvard University	66
4	6	Matsue Y	3	0.00	Juntendo University	20
5	6	Narula J	376	0.02	Icahn School of Medicine at Mount Sinai	88
6	5	Matsumoto S	3	0.00	Japanese Circulat Soc	27
7	5	Yonetsu T	3	0.00	Tokyo Medical & Dental University	27
8	5	Kohsaka S	3	0.00	Keio University	32
9	5	Kitai T	3	0.00	National Cerebral & Cardiovascular Center–Japan	24
10	5	Wang W	68	0.00	Huazhong University of Science & Technology	43

BC, Betweenness Centrality.

**Figure 4:**
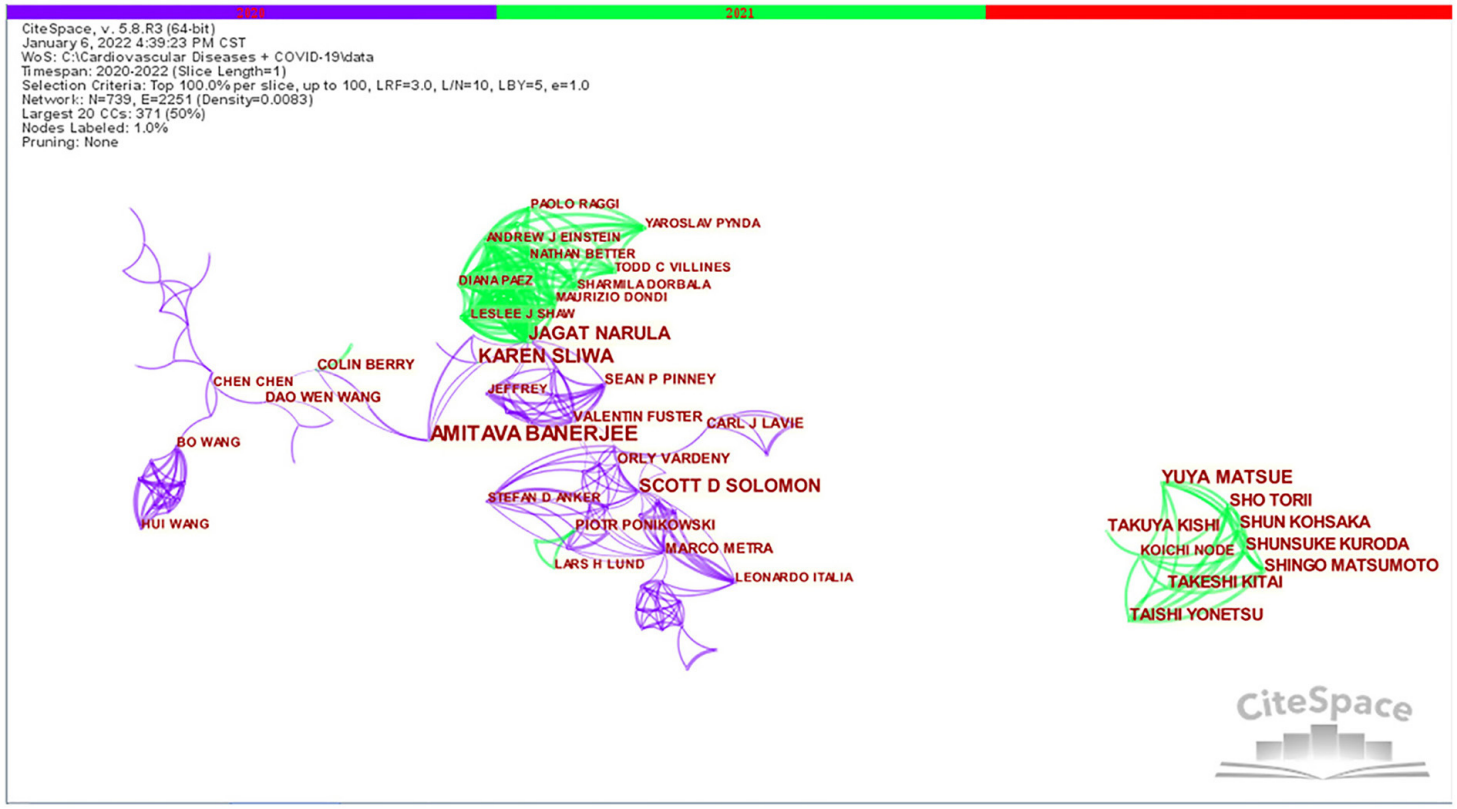
Visualization map of authors in the studies of CVDs and COVID-19 research.

Figure 4 shows that there is a cooperative network between different authors. Each node represents an author, the connections represent the existence of a cooperative relationship between the authors, the color of the connections indicates the first instance of cooperation, and the thickness of the connection represents the intensity of cooperation between authors. As shown in Figure 4, Banerjee A, Narula J, Sliwa K, Pablo P, and Harry H worked closely together. Yonetsu T worked closely with Matsue Y, Kohsaka S, Takuya K, and Kitai T. In addition, Solomon SD, Leon A, Orly V, Stefan DA, and Marco M worked closely together.

### Distribution of journals and co-cited journals

**Figure 5:**
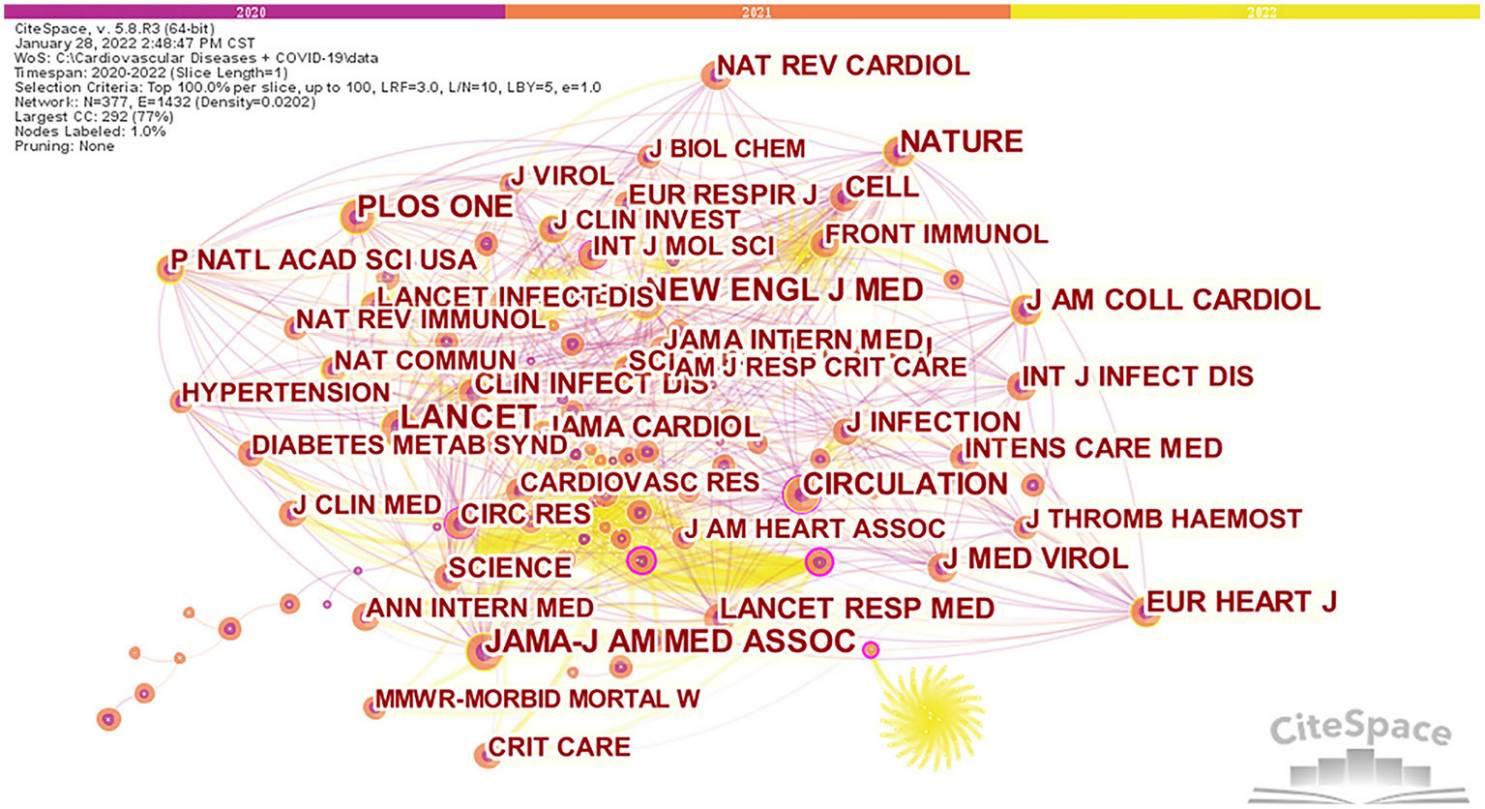
Network map for citation analysis of co-cited journals on CVDs and COVID-19 research.

Figure 5 shows the network visualization maps for citation analysis of journals that are active in CVD and COVID-19 research. We found that 1,699 publications related to CVDs and COVID-19 research were published in 737 journals. *Frontiers in Cardiovascular Medicine* (33, 1.98%) had the highest number of publications, followed by *Journal of Clinical Medicine* (31, 1.86%). Among the top 10 journals, *Journal of the American College of Cardiology* (24.09) had the highest impact factor (IF).

Furthermore, Table 4 shows that 70% of journals belong to Q1. The influence of a journal is determined by the number of times the journal is co-cited, which reflects whether the journal has significant influence in a particular research field (40). Among the top 10 co-cited journals, three journals have been cited more than 1,000 times. As shown in Table 4, the journal with the highest number of citations is *The Lancet* (1,190), followed by *New England Journal of Medicine* (1,083). According to the 2020 Journal citation report (JCR), almost all of the co-cited journals in the top 10 journals were in the Q1 region, except for *Plos One*.

**Table 4:** Top 10 journals and co-cited journals in CVDs and COVID-19 research.

**Rank**	**Journal**	**Count** **(% of 1,669)**	**IF (2020)**	**JCR**	**co-cited journal**	**Citations**	**Degree**	**BC**	**IF (2020)**	**JCR**
1	*Frontiers in Cardiovascular Medicine*	33(1.98%)	6.05	Q1	*Lancet*	1,190	36	0.06	79.32	Q1
2	*Journal of Clinical Medicine*	31(1.86%)	4.24	Q1	*New England Journal of Medicine*	1,083	35	0.02	91.25	Q1
3	*International Journal of Environmental Research and Public Health*	28(1.68%)	3.39	Q2/Q2/Q1	*Journal of the American Medical Association*	1,019	31	0.02	56.27	Q1
4	*Plos one*	28(1.68%)	3.24	Q2	*Circulation*	732	32	0.16	29.69	Q1/Q1
5	*International Journal of Molecular Sciences*	22(1.32%)	5.92	Q1/Q2	*Plos One*	625	29	0.03	3.24	Q2
6	*Journal of the American Heart Association*	19(1.14%)	5.50	Q1	*Nature*	576	34	0.04	49.96	Q1
7	*Scientific Reports*	17(1.02%)	4.38	Q1	*JAMA Cardiology*	554	23	0.01	14.68	Q1
8	*Journal of the American College of Cardiology*	16(0.96%)	24.09	Q1	*European Heart Journal*	551	22	0.01	29.98	Q1
9	*Medical Hypotheses*	15(0.90%)	1.54	Q4	*British Medical Journal*	546	18	0.21	14.09	Q1
10	*Global Heart*	14(0.84%)	3.43	Q2	*Lancet Respiratory Medicine*	538	26	0.07	30.70	Q1

BC, Betweenness Centrality.

### Co-cited references

Co-citation analysis involves tracking two or more references that are cited in the same literature (41). By analyzing the clusters and turning points in the co-citation network, the knowledge structure of a research area and its transformations can be revealed (42). From the 389 co-cited references retrieved, the top 10 co-cited references are shown in Table 5, of which “Clinical course and risk factors for mortality of adult inpatients with COVID-19 in Wuhan, China: a retrospective cohort study” (43) was the most frequently cited (523).

**Table 5:** Top 10 most cited references of publications in CVDs and COVID-19 research.

**Rank**	**Article title**	**Frequency**	**BC**	**Reference**
1	Clinical course and risk factors for mortality of adult inpatients with COVID-19 in Wuhan, China: a retrospective cohort study	523	0.16	(43)
2	Clinical characteristics of coronavirus disease 2019 in China	373	0.03	(44)
3	Clinical features of patients infected with 2019 novel coronavirus in Wuhan, China	344	0.03	(45)
4	Cardiovascular implications of fatal outcomes of patients with coronavirus disease 2019 (COVID-19)	284	0.04	(20)
5	Association of cardiac injury with mortality in hospitalized patients with COVID-19 in Wuhan, China	278	0.02	(46)
6	Characteristics of and important lessons from the coronavirus disease 2019 (COVID-19) outbreak in China summary of a report of 72 314 cases from the Chinese center for disease control and prevention	261	0.00	(47)
7	Epidemiological and clinical characteristics of 99 cases of 2019 novel coronavirus pneumonia in Wuhan, China: a descriptive study	236	0.01	(48)
8	SARS-CoV-2 cell entry depends on ACE2 and TMPRSS2 and is blocked by a clinically proven protease inhibitor	231	0.03	(49)
9	Clinical course and outcomes of critically ill patients with SARS-CoV-2 pneumonia in Wuhan, China: a single-centered, retrospective, observational study	206	0.01	(50)
10	COVID-19 and the cardiovascular system	191	0.00	(51)

BC, Betweenness Centrality.

**Figure 6:**
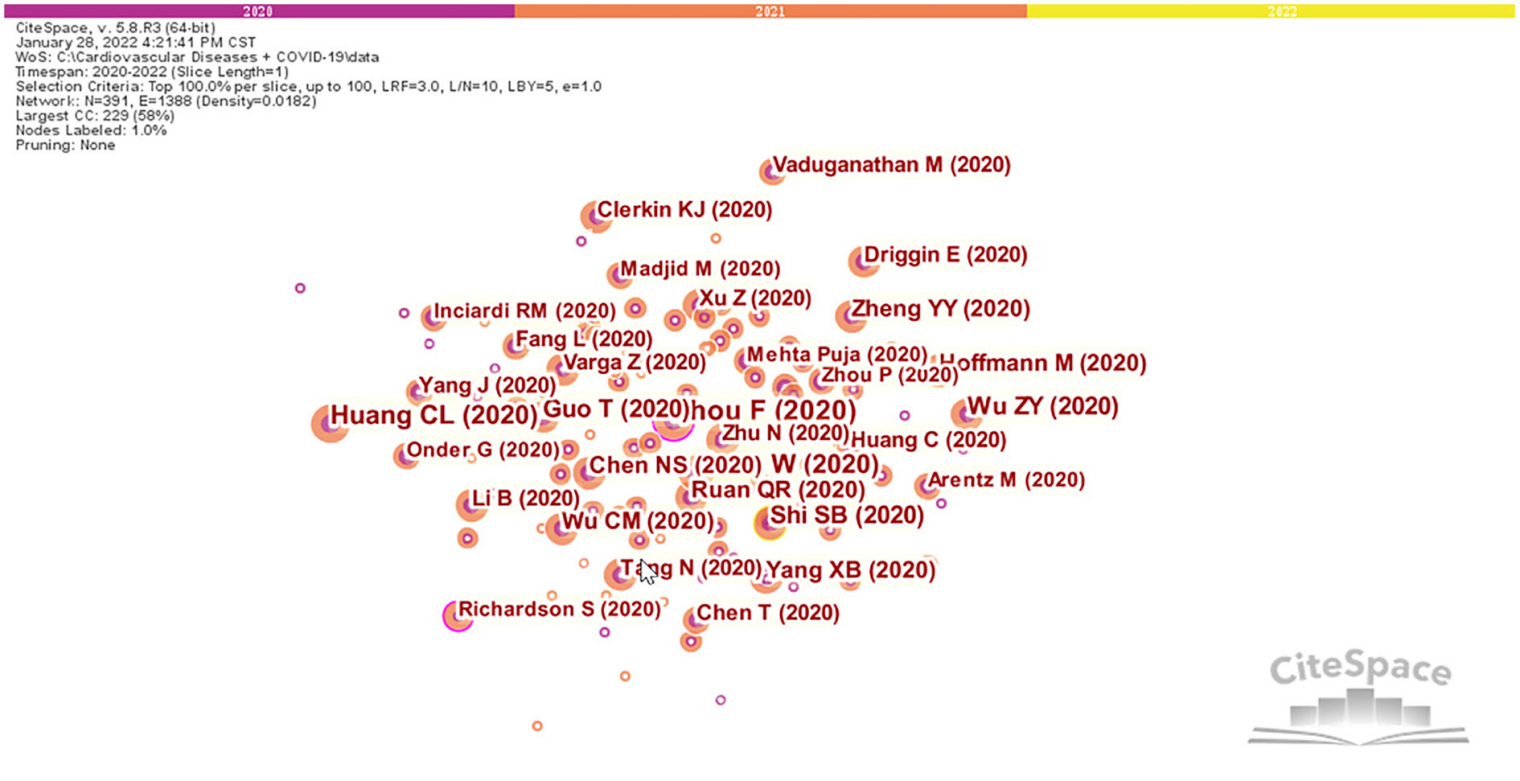
References co-citation network in the studies of CVDs and COVID-19 research.

Figure 6 shows the co-citation network for CVDs and COVID-19 research. Two nodes can be clearly seen with a purple circle in the network. The betweenness centrality of “Clinical course and risk factors for mortality of adult inpatients with COVID-19 in Wuhan, China: a retrospective cohort study” is 0.16 (43). That paper reviewed the relevant literature and it was published online in the *Lancet* in March 2020. The study presented details of 191 laboratory-confirmed COVID-19 patients admitted to Wuhan Jinyintan Hospital and Wuhan Pulmonary Hospital before January 31, 2020. It was the largest retrospective cohort study conducted at that time among COVID-19 patients who experienced definitive symptoms (43). The results showed that the potential risk factors for early identification of patients with poor prognosis included old age, high sequential organ failure assessment (SOFA) score and D-dimer > 1 μg/L (43). Prolonged viral shedding provides a theoretical basis for future isolation of infected patients and optimal antiviral interventions (43). The study found, for the first time, that patients had developed sepsis without bacterial infection. It was revealed that the median time for detoxification of COVID-19 in surviving patients was 20 days, and the longest duration of virus detoxification was 37 days (43). This also partly explains why some patients have nucleic acid reactivation after discharge.

From the second node, the betweenness centrality of the article “Presenting Characteristics, Comorbidities, and outcomes among 5,700 patients hospitalized with COVID-19 in the New York City Area” is 0.19 (52). That article was published in *JAMA-Journal of the American Medical Association* in April 2020 and it was the first large-scale case study in USA to include COVID-19 inpatients (52). By analyzing the characteristics and early results of 5,700 inpatients with COVID-19 in New York, it was found that the most common complications were hypertension, obesity, and diabetes (52).

### Research hotspots and frontiers analysis

#### Research hotspots analysis

**Figure 7:**
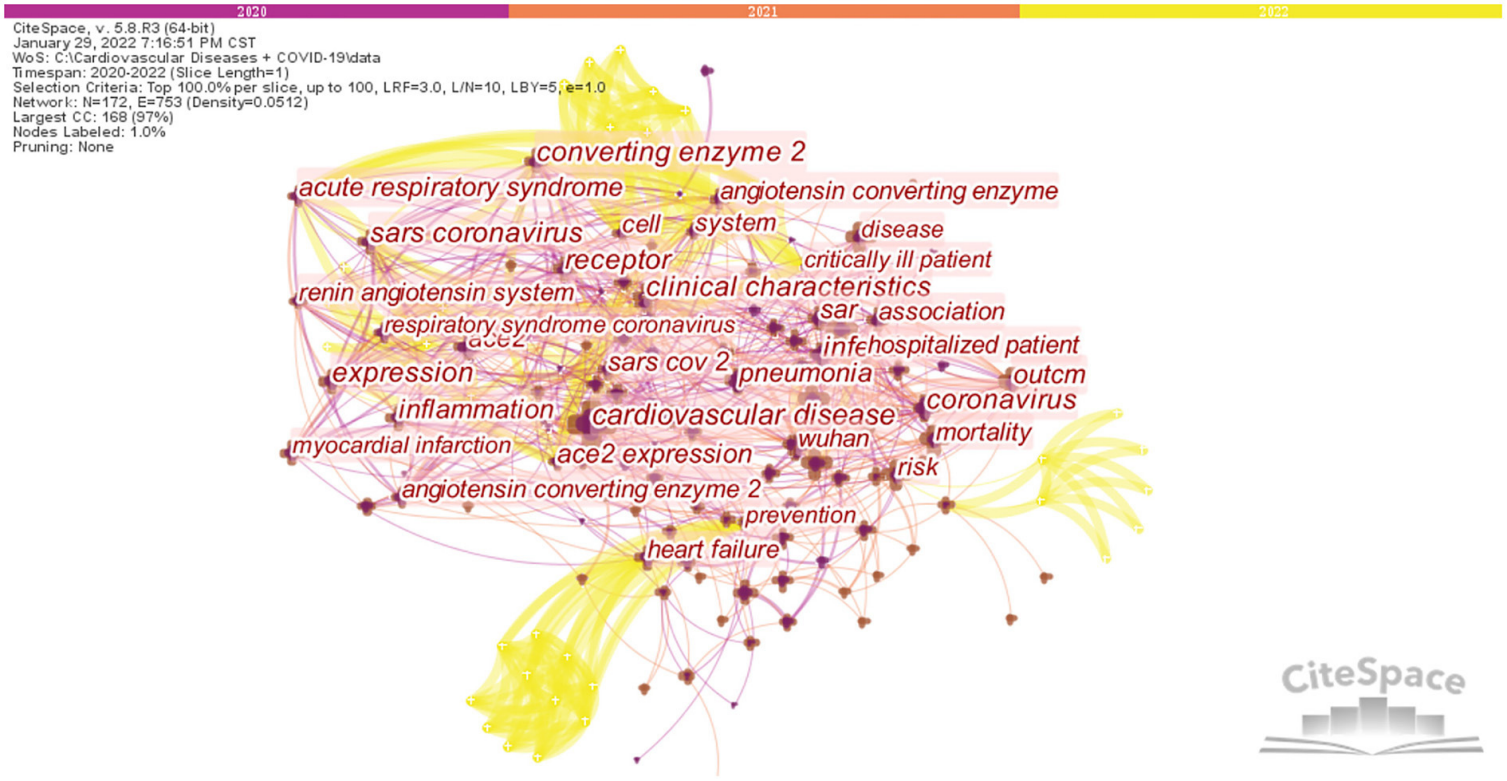
Network of the main keywords in the studies of CVDs and COVID-19 research.

Keywords are the core and essence of a paper. Co-word analysis uses the co-occurrence of word pairs or noun phrases in a collection to determine the relationships between topics in the discipline represented by the collection (53). Figure 7 displays the co-occurrence analysis results for keywords in CVD and COVID-19 research. A network was obtained with 172 nodes, 753 connections and a density of 0.0512, Modularity Q = 0.5234, and Silhouette S = 0.7579. After running the software, synonymous keywords were merged to obtain the top 20 keyword co-occurrence networks in the research literature, and the results are shown in Table 6. For example, COVID 19 was merged with coronavirus disease 2019 and coronavirus disease 2019 (COVID-19) to form COVID-19; and ace2, angiotensin converting enzyme 2, and angiotensin-converting enzyme 2 were merged into ACE2. Besides cardiovascular disease (441) and COVID-19 (253), other keywords with high frequency in this study include ACE2 (192), mortality (122), risk (118), coronavirus (111), infection (110), Wuhan (108), pneumonia (106), and risk factor (102). These keywords reflect the popular research hotspots in this field. As shown in Table 6, the specific diseases involved include cardiovascular disease, COVID-19, pneumonia, heart failure, myocardial infarction, and acute respiratory syndrome.

**Table 6:** Top 20 keywords related to CVDs and COVID-19 research.

**Rank**	**Keyword**	**Frequency**	**Degree**	**BC**	**Rank**	**Keyword**	**Frequency**	**Degree**	**BC**
1	Cardiovascular disease	441	31	0.33	11	Clinical characteristics	97	23	0.03
2	COVID-19	253	43	0.08	12	Receptor	88	23	0.06
3	ACE2	192	42	0.09	13	Heart failure	82	17	0.21
4	Mortality	122	17	0.09	14	Expression	80	23	0.16
5	Risk	118	18	0.2	15	Inflammation	74	19	0.15
6	Coronavirus	111	26	0.08	16	Myocardial infarction	72	14	0.08
7	Infection	110	23	0.07	17	SARS coronavirus	71	25	0.07
8	Wuhan	108	17	0.02	18	Acute respiratory syndrome	56	20	0.04
9	Pneumonia	106	19	0.04	19	Management	55	8	0.03
10	Risk factor	102	7	0.01	20	Oxidative stress	48	10	0.03

BC, Betweenness Centrality.

**Figure 8:**
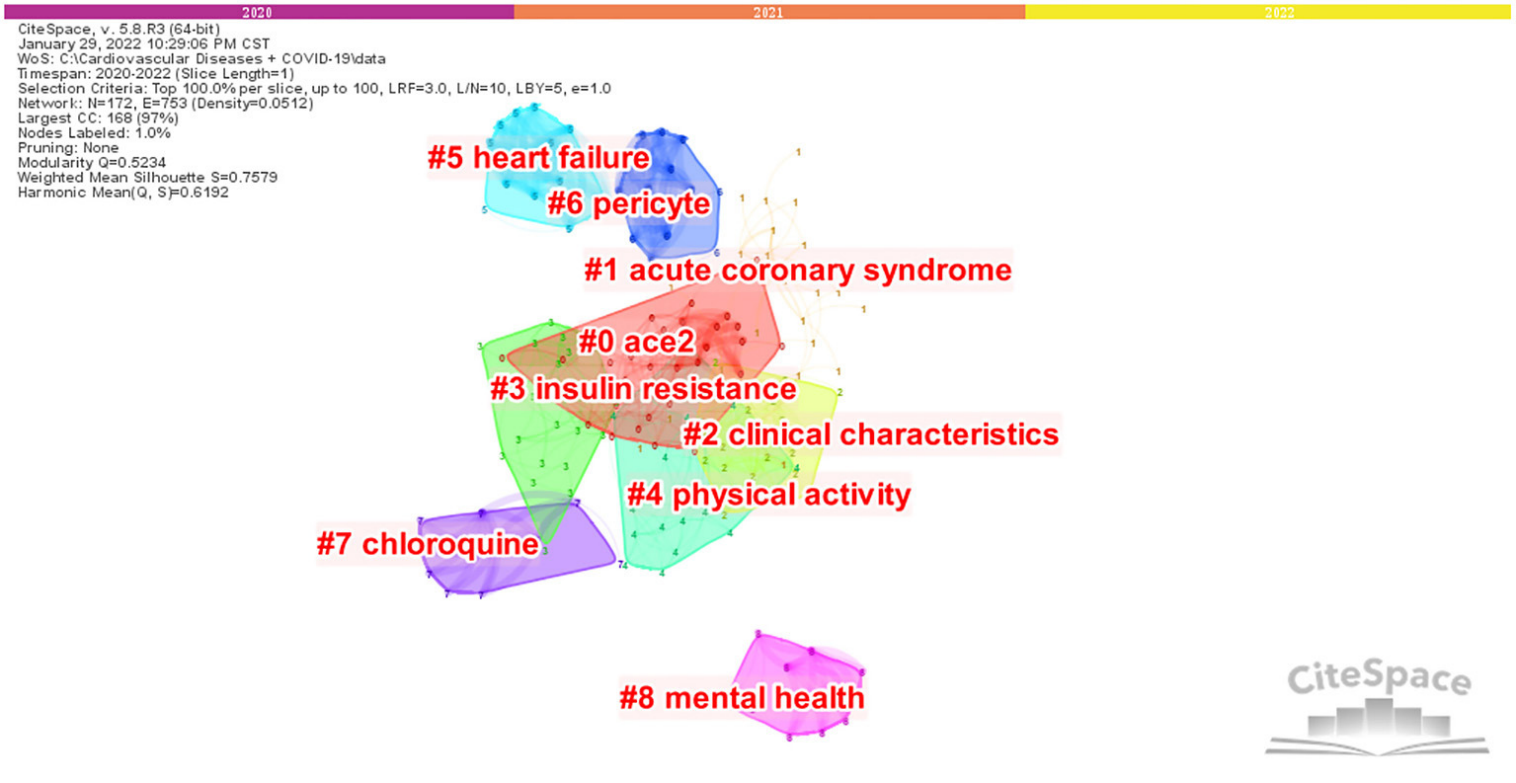
Network of the main keywords' clusters in the studies of CVDs and COVID-19 research.

Based on keywords co-occurrence analysis, clusters can be formed in the network map to summarize the research hotspots and obtain the basic knowledge structure. Figure 8 shows nine clusters of keywords, with each cluster composed of multiple closely related words. These nine clusters are #0 ACE2, #1 acute coronary syndrome,#2 clinical characteristics, #3 insulin resistance, #4 physical activity, #5 heart failure, #6 pericyte, #7 chloroquine, and #8 mental health.

#### Identification of research frontiers

**Figure 9:**
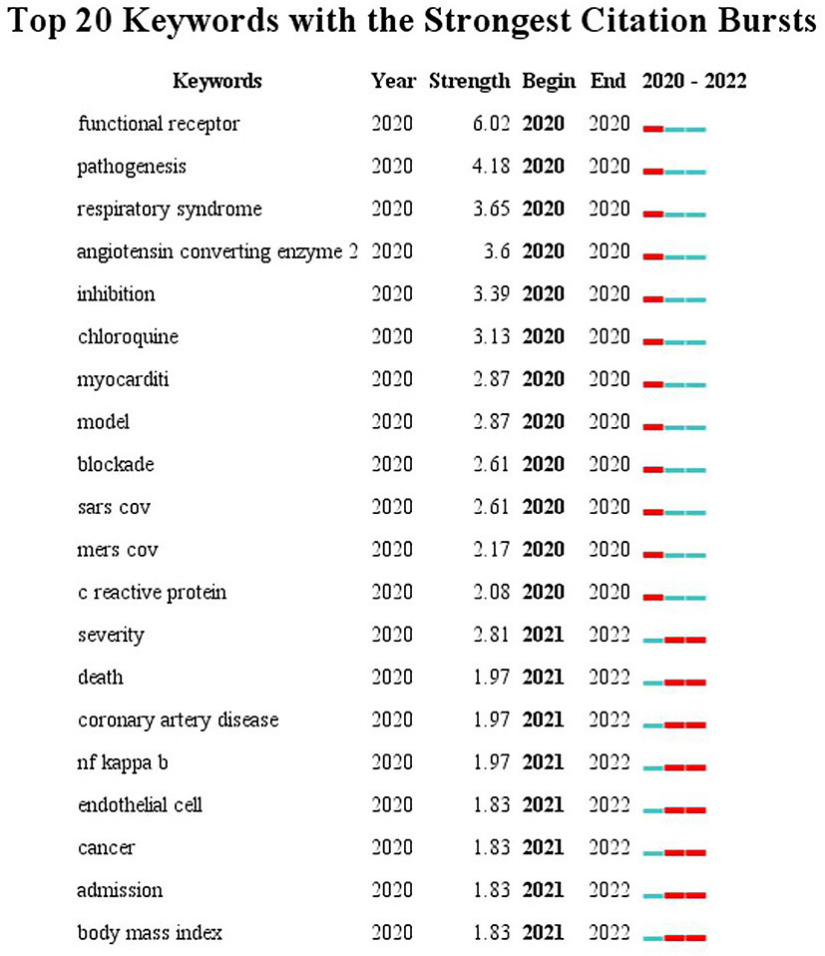
Top 20 Keywords with the Strongest Citation Bursts in the studies of CVDs and COVID-19 research.

The burst detection provided by CiteSpace can extract emergent words among subject words in the literature to clearly show the research frontiers and development trends of a certain discipline (54). In this study, keywords burst detection was conducted based on the keywords co-occurrence network, and the top 20 keywords with the strongest citation bursts in CVDs and COVID-19 research were reported. As illustrated in Figure 9, the blue line denotes the time axis and the red segment on the blue time axis displays the burst detection, indicating the start year, end year, and burst duration. The top keywords with the strongest citation bursts can reflect emerging academic trends and new hotspots, predict research frontiers, and explore potential popular topics in CVDs and COVID-19 research (55). Notably, “functional receptor” had the strongest citation bursts, with a maximum burst strength of 6.02, followed by “pathogenesis” and “respiratory syndrome” at 4.18 and 3.65, respectively. The next 17 top keywords with the strongest citation bursts were: angiotensin converting enzyme 2, inhibition, chloroquine, myocarditis, model, blockade, SARS-CoV, MERS-CoV, c reactive protein, severity, death, coronary artery disease, NF-κB, endothelial cell, cancer, admission, and body mass index.

## Discussion

### General information

Given the number of publications and distribution types in CVDs and COVID-19 research, the results show that there is greater on clinical practice and specific scientific research. However, in the wake of the COVID-19 pandemic, global attention is more focused on the impact of the disease on public health. Various countries have invested a lot of manpower and material resources to study the disease and address the impacts. Therefore, many high-quality studies have been published in a short period of time.

By analyzing the results of countries/regions and institutions, it was found that USA, China, Italy, and England are the countries with the largest number of publications in this field. In fact, this finding is related to the outbreak of the pandemic starting in Wuhan, China, and then spreading around the world, with England, Italy, USA, and other European countries becoming the hardest hit areas. America and European countries have developed economies, a high level of medical technology, and strong data management and academic systems. These factors have contributed to the high number of relevant studies from these countries. In addition, through the visual knowledge maps in Figures 2, 3, we can clearly see that the links and cooperation between various countries and institutions are relatively close, which is conducive to creating global strength to solve the COVID-19 pandemic and the problems it brings.

The visual analysis of institutions reveals that Harvard Medical School not only ranked first in terms of the number of published papers, but the quality of their CVDs and COVID-19 research was also very high. In fact, according to the relevant literature, Harvard Medical School was also investigating the relationship between CVDs and COVID-19, including summarizing the potential impacts of coronavirus on the cardiovascular system (55). The researchers at Harvard Medical School believe that COVID-19 is associated with a high inflammatory burden, and they are working to find specific vaccines and antiviral drugs against SARS-CoV-2 (56). Their specific projects include: participating in a study comparing the clinical status of patients with moderate COVID-19 on day 11 treated with Remdesivir vs. standard therapy (57); explaining why COVID-19 disproportionately affects the elderly from the perspective of molecular differences and examining treatment methods to improve the survival of the elderly (58); pharmacoepidemiologic studies to clarify whether renin-angiotensin system (RAS) inhibition mediates the relationship between CVDs and COVID-19 mortality (59); and an investigation of the role of cardiac injury and HF in COVID-19, its pathogenesis and potential therapeutic implications (60). Longitudinal trends in hospitalizations for acute cardiovascular disease in the tertiary health care system were studied and it was found that in the first phase of the COVID-19 pandemic, the rate of hospitalizations for acute cardiovascular disease decreased significantly and the length of hospital stay for hospitalized patients was shortened (61). These data confirm that acute care for CVDs may be delayed or shortened during the COVID-19 pandemic (61). A multicentre, observational cohort study evaluating the frequency, risk factors, prevention, and management patterns of arterial and venous thromboembolic disease in patients with COVID-19 concluded that despite high thromboprophylaxis use, major arterial or venous thromboembolism, major adverse cardiovascular events and symptomatic venous thromboembolism were more common in patients with COVID-19, especially in the intensive care setting (62). These have been important questions for CVDs patients and physicians since the beginning of the COVID-19 pandemic. The publication of these documents will help people better understand and solve the current problems.

The evaluation criteria for core authors include the number of published documents, the total citations, and h-index (63). Analysis of the core authors is helpful for exploring the distribution of the publications. Based on an analysis of the relevant literature, Professor Banerjee A from the University of London, UK is the author with the most frequent occurrence in this field. Banerjee A's main research direction is the clinical research of COVID-19 in conjunction with CVDs, including: management of patients with CVDs diagnosed or suspected of COVID-19 under limited resource conditions (64); description of cardiovascular manifestations and cardiovascular risk factors in hospitalized COVID-19 patients (65); modeling studies to estimate the number of individuals at increased risk of severe COVID-19 due to underlying health conditions globally, regionally and nationally during 2020 (66); a retrospective cohort study comparing the rate of organ-specific dysfunction in COVID-19 patients after discharge with a matched control group from the general population (67); assessing the impact of the COVID-19 pandemic on cancer care services and overall excess deaths among cancer patients (68); monitoring hospital CVDs presentation, diagnosis, and treatment activities during the COVID-19 pandemic to understand indirect effects (69); using electronic health records for primary care in England to investigate the prevalence, incidence, and outcomes of a range of specific CVDs among homeless people (70); as well as population-wide studies of COVID-19 and CVDs (71).

Professor Solomon SD from Harvard University is the author with the highest number of citations. Solomon SD mainly focuses on researching the mechanism of the disease, including some clinical studies. His specific studies include: the potential effects of coronavirus on the cardiovascular system (56); the cardiovascular effects of COVID-19 and influenza infection (72); description of the role of cardiac injury and HF in COVID-19, its pathogenesis and potential therapeutic significance (60); description of the impact of influenza vaccination on cardiovascular morbidity and mortality among COVID-19 patients (73) and longitudinal trends in tertiary health system hospitalizations for acute cardiovascular disease during the COVID-19 pandemic (61).

Professor Narula J from the Icahn School of Medicine at Mount Sinai has the highest h-index. Narula J's research directions are: clinical research, discussion of policy guidelines, and research on disease mechanisms. His specific studies include the incidence and impact of myocardial injury in patients hospitalized with COVID-19 infection (74), the international impact of COVID-19 on cardiac diagnosis (75), and the impact on cardiovascular testing in USA and the rest of the world (76). In addition, Narula J also elaborated on the clinical significance of the interactions between SARS-Cov2 and the renin-angiotensin system (77), and he conducted a special workshop on coronavirus and cardiometabolic syndrome (78).

Visualized knowledge maps can provide information on influential research teams and potential collaborators, and help researchers build collaborative relationships (38). In the context of the COVID-19 pandemic, these representative authors have made important contributions to the knowledge base and discussion of theoretical mechanisms, clinical case studies, and their impact on various countries and regions. These authors have a strong academic reputation in this field.

According to the top 10 journals shown in Table 4, the journal with the most articles on CVDs and COVID-19 is *Frontiers in Cardiovascular Medicine*, followed by *Journal of Clinical Medicine* and *International Journal of Environmental Research and Public Health*. According to the journal ranking, the main research focus in this field is currently the specific clinical problems and public health problems involved since the outbreak of COVID-19, which will also be the key research direction in this field in the future. As for co-cited journals, *The Lancet* was the most cited journal, followed by *New England Journal of Medicine* and the *Journal of the American Medical Association*. Analyzing the distribution of publication sources can be helpful for revealing the core journals. The co-cited journals are from high-impact journals, indicating that CVDs and COVID-19 research is highly valued in the global academic community.

Among the top 10 co-cited references, 8 articles were about the clinical characteristics and clinical course of COVID-19, which shows that people in this field are paying more attention to the actual clinical situations of patients, which is also in line with the specific situation we are currently facing. In addition, we also obtained two articles with betweenness centrality > 0.1. These two documents (43, 52) can be considered as landmark events in this field and they have far-reaching impact on the development of this field. In fact, these two articles have been cited more than 2,000 times, of which “Clinical course and risk factors for mortality of adult inpatients with COVID-19 in Wuhan, China: a retrospective cohort study” (43), ranked fifth in the world in 2020, with a cumulative citation frequency of 4,881.

### The hotspots and frontiers

#### The hotspots in CVDs and COVID-19

Based on the results of keyword clustering and the top 20 keywords with the strongest citations, the current research hotspots in CVDs and COVID-19 are indicated by these nine clusters. From these nine clusters, we can see that the specific diseases involved in the basic knowledge structure for this field were #1 acute coronary syndrome and #5 heart failure. The pathogenesis of the disease was #0 ACE2, #3 insulin resistance, and #6 pericyte. The specific therapeutic drugs were #7 chloroquine and #2 clinical characteristics were #4 physical activity and #8 mental health. #0 ACE2 was the largest cluster and the main research hotspot for CVDs and COVID-19 research. In the next sections, we will analyze the content of these clusters in detail.

First, the specific disease involved was #1 acute coronary syndrome. According to the relevant literature, patients with acute coronary syndrome (ACS) infected with SARS-CoV-2 were generally in serious condition, with high mortality and poor prognosis (79). During the COVID-19 pandemic, the European Society of Cardiology Guidelines for the Diagnosis and Management of Cardiovascular Disease also noted that COVID-19 inpatients often showed signs of myocardial damage upon admission, possibly because many ACS patients did not receive medical care and hospitalization for fear of contracting COVID-19. In addition, several studies have suggested that plaque instability and supply-demand imbalance were key causes of COVID-19 induced ACS (80). #5 heart failure was identified as a risk factor for a more severe course of COVID-19 and increased mortality (80). In particular, acute HF may complicate the clinical course of COVID-19, especially in severe cases. Patients with chronic HF may be at higher risk for COVID-19 due to their older age, multiple comorbidities, and a significantly higher risk of adverse outcomes. A meta-analysis by Sentongo et al. concluded that COVID-19 patients with HF had a higher risk of death compared to those with coexisting conditions (81). Tomasoni et al. believe that patients with heart failure are more susceptible to COVID-19 and, once infected, have a more severe clinical course (60). Studies have shown that at least 10% of hospitalized COVID-19 patients developed HF and myocardial damage, and the proportion can be 25–35% or higher when critical patients or those with heart disease were considered (60).

Second, specific disease mechanisms of COVID-19 include #0 ACE2, which is a membrane-bound aminopeptidase that was discovered in 2000 (82, 83). ACE2 is a key molecular target for the occurrence and development of COVID-19. The surface spike protein of SARS-CoV-2 binds to ACE2 and enters human cells (4). On the one hand, it will cause a series of pathological reactions that support the expression and replication of SARS-CoV-2 and destroy the human body (84). On the other hand, by lowering the level of ACE2, the ACE2/Ang (1–7)/Mas receptor pathway will be inhibited, breaking the balance of renin angiotensin aldosterone system (RAAS), which is not conducive to the maintenance of hemodynamic stability and normal cardiorenal function, and may induce acute myocardial injury, ACS, arrhythmia, and HF, leading to increased mortality of COVID-19 patients (45, 85). Angiotensin converting enzyme inhibitor (ACEI) and angiotensin receptor antagonist (ARB) not only inhibit the classical RAAS pathway, but they also up-regulate the level of ACE2. Therefore, a large amount of pharmacoepidemiologic evidence is needed to support the use of ACEI/ARB during COVID-19 prevention and control (59, 77, 86). Gender and age differences in ACE2 gene expression and immune responses have also been suggested to be important reasons for reduced susceptibility to COVID-19 infection and improved survival in women (87). #3 insulin resistance can lead to neurodegeneration, HF and viral infection (88). Insulin resistance is an important pathophysiological cause of poor prognosis in COVID-19 patients (89). Studies have shown increased coagulopathy and ACE2 expression in adipose cells and pulmonary adipose fibroblasts in insulin-resistant patients. This process enhances SARS-CoV-2 binding and cell uptake and drives a high immune response, leading to pulmonary fibrosis and multi-organ system failure (90–92). #6 cardiac pericytes highly express ACE2, and therefore may be a direct target of SARS-CoV-2 (93). Studies have shown that pericytes may be a highly infectious cell population of SARS-CoV-2. They may promote the imbalance between ANGPT1/2 and TIE2, disrupt the integrity of the vascular barrier and increase vascular permeability, leading to endothelial dysfunction and participation in the occurrence and development of hypertension (93). They also induce dysfunction of myocardial microcirculation (94, 95). Pericytes play an important role in the microvascular system, and infection of pericytes may impair the ability of the myocardial supply to meet metabolic needs (96). Patients with underlying CVDs have higher ACE2 expression levels in pericytes and they experience more severe disease (95).

A specific drug involved in this study is #7 chloroquine. The antimalarial drugs chloroquine and hydroxychloroquine were originally authorized by the FDA for emergency use (EUA) and was used in many COVID-19 treatment regimens (96, 97). The mechanism of this drug is that it can induce antiviral activity against many RNA viruses, including SARS and SARS-CoV-2, by increasing endosomal pH and interfering with glycosylation processes (98). At the time of the COVID-19 outbreak, a clinical study demonstrated the clinical benefits of chloroquine in patients with COVID-19 compared to control treatments (99). However, after more clinical studies, it was found that chloroquine lacks clinical efficacy and has obvious side effects, especially adverse cardiac events (100). The FDA withdrew the EUA on May 28, 2021 (101). Hence, this cluster has attracted extensive attention of scholars and is a much-researched topic.

In addition, there are #2 clinical characteristics that describe the clinical characteristics of different types of COVID-19 patients from different regions, different comorbidities, different age groups, vaccinated with different types of vaccines or unvaccinated. These characteristics are of great value for early intervention and later improvement of COVID-19 treatment plans. For example, Huang C et al. introduced the epidemiological, clinical, laboratory diagnostic and imaging characteristics, treatment, and clinical prognosis of 41 COVID-19 patients (45). Guan WJ et al. systematically collected 1,099 laboratory-confirmed COVID-19 cases from 552 hospitals in 30 provinces from December 2019 to January 29, 2020, and described the clinical characteristics of all cases in detail (102). Wang D et al. described in detail the epidemiological, demographic, clinical, laboratory, and radiological characteristics and treatment of 138 hospitalized COVID-19 patients (11). Chen N et al. analyzed epidemiological, demographic, clinical, radiographic, and laboratory data from nine COVID-19 cases and reported the clinical outcomes at follow-up (48).

As for #4 physical activity, it is without doubt that the COVID-19 pandemic has changed our way of life, and the reduction in physical activity due to lockdown measures may also lead to failure to control cardiovascular risk factors (103). Therefore, experts suggest that people should engage more frequently in physical activities, whether at home or in an outdoor area, which not only improves people's level of happiness, but also reduces cardiovascular risk factors, such as maintaining physical activity to avoid venous thromboembolism (104). Studies have shown that physical activity trackers can significantly increase physical activity and may be a useful auxiliary tool for promoting a healthy lifestyle (105).

#8 mental health studies have shown that COVID-19 lockdowns cause significant psychological effects on people, including: greater psychological stress, post-traumatic stress disorder (PTSD) symptoms, depressive symptoms, more severe anxiety, insomnia, and irritability (106). Stress caused by COVID-19, economic depression and isolation are also likely to lead to a decline in mental health. Furthermore, lower socioeconomic status was associated with an increased risk of CVDs mortality and CVDs risk factors (107).

#### The frontiers in CVDs and COVID-19 research

Based on keyword emergence in the literature, we speculate that ACE2 and NF-κB are the research frontiers and potential hotspots in this field. ACE2 is a functional receptor of SARS-CoV-2 (86). SARS-CoV-2 uses ACE2 as a functional receptor to enter human cells (108), causing the endocytosis of the ligand/receptor complex, inducing fusion between the virus and host cells, and then ACE2 is degraded in the cells. NF-κB is a complex protein system that exists in the cytoplasm. NF-κB signaling pathway is a typical pro-inflammatory pathway that plays a key role in the functions of the immune system and mediates a variety of cellular processes, including immune response, inflammation, cell proliferation, and cell survival (109, 110). Studies have shown that strategies can be developed to mitigate the cytokine storm and reduce the pathology of severe COVID-19 by focusing on how the NF-κB signaling pathway modulates the inflammatory response (111). Furthermore, identifying potential therapeutic targets related to the NF-κB signaling pathway could help control the more severe spectrum and mortality associated with the pandemic (112). A study by Hamid T et al. showed that chronic activation of NF-κB can lead to vascular disturbance and cardiomyocyte remodeling in patients with metabolic syndrome, which predisposes them to cardiac complications (113). NF-κB signaling pathway also plays an important role in COVID-19 drug treatments. Studies have shown that remdesivir reduces cytokine storm and severe diseases by reducing dsRNA-related induction of the NF-κB signaling pathway (114). Hydroxychloroquine blocks NF-κB signaling pathway by reducing levels of TNF-α, TNF-1β, IgG, and IFN-γ (115). Monoclonal antibodies such as Infliximab and Adalimumab inhibit atypical activation of NF-κB pathway by reducing TNF-α levels, which has the potential to relieve symptoms in critically ill patients with COVID-19 (116). In summary, inhibition of NF-κB pathway has a potential therapeutic role in alleviating severe forms of COVID-19. NF-κB inhibition may be one of the mechanisms of action of currently effective in anti-COVID-19 drugs. More work is needed to identify direct NF-κB inhibition as a treatment for severe forms of this disease (112). Therefore, we believe that ACE2 and NF-κB will be the focus of future research.

### Limitations

This study has some limitations. First, the analysis of this study is based on articles from the Web of Science Core Collection database, which is the most well-known database of scientific publications on many research topics, but other databases such as PubMed, Scopus may provide broader coverage. Second, this article only includes English articles, which reduces the number of articles that were retrieved. Finally, the literature on CVDs and COVID-19 is overwhelming. With the rapid updating of research hotspots and frontiers, we may miss some research hotspots. However, visual analysis based on literature lays a foundation for understanding the research topics and frontiers in CVDs and COVID-19 research.

## Conclusion

This study provides useful information for the research of CVDs and COVID-19, including potential collaborators, popular research topics, and a reference for more extensive and in-depth research in the future. Currently, research on CVDs and COVID-19 is in a period of rapid development, which indicates that this field has attracted more and more attention. Our findings are a summary of the current state of CVDs and COVID-19 and have important implications for future research direction.

## Data availability statement

The raw data supporting the conclusions of this article will be made available by the authors, without undue reservation.

## Author contributions

NW and YX designed the study and wrote the manuscript. HW, QJ, and XS collected the data. HW and XZ re-examined the data. NW, NZ, and YL analyzed the data. HZ and YH reviewed and revised the manuscript. All authors contributed to the article and approved the submitted version.
